# Opportunities and challenges to integrating mental health into HIV programs in a low- and middle-income country: insights from the Nigeria implementation science Alliance

**DOI:** 10.1186/s12913-020-05750-0

**Published:** 2020-09-29

**Authors:** Echezona E. Ezeanolue, Theddeus Iheanacho, Isaac A. Adedeji, Ijeoma Uchenna Itanyi, Babayemi Olakunde, Dina Patel, Patrick Dakum, Prosper Okonkwo, Timothy Akinmurele, Michael Obiefune, Hadiza Khamofu, Bolanle Oyeledun, Muyiwa Aina, Andy Eyo, Obinna Oleribe, John Oko, Ayodotun Olutola, Ibrahim Gobir, Muktar H. Aliyu, Gambo Aliyu, Godfrey Woelk, Gregory Aarons, George Siberry, Rachel Sturke, Nigeria Implementation Science Alliance

**Affiliations:** 1 Center for Translation and Implementation Research, Institute of Maternal and Child health, University of Nigeria Nsukka, Enugu Campus, Enugu, Nigeria; 2 Healthy Sunrise Foundation, Las Vegas, NV USA; 3 Department of Psychiatry, Yale University School of Medicine, Yale University, 300 George Street, New Haven, CT 06511 USA; 4 Olabisi Onabanjo University, Ago-Iwoye, Nigeria; 5 Department of Community Medicine, University of Nigeria, Enugu, Nigeria; 6 National Agency for Control of AIDS, Abuja, Nigeria; 7 Institute of Human Virology, University of Maryland, Baltimore, MD USA; 8 Institute of Human Virology, Abuja, Nigeria; 9 APIN Prevention Initiative, Abuja, Nigeria; 10 Enhanced Health Access Initiatives, Abuja, Nigeria; 11 University of Maryland, Baltimore, MD USA; 12 Family Health International, Abuja, Nigeria; 13 Center for Integrated Health Programs, Abuja, Nigeria; 14 Solina Health, Abuja, Nigeria; 15 Excellence Community Education Welfare Scheme, Abuja, Nigeria; 16 Excellence and Friends Management Consult, Abuja, Nigeria; 17 Catholic Caritas Foundation Nigeria, Abuja, Nigeria; 18 Centre for Clinical Care and Clinical Research, Abuja, Nigeria; 19 Center for Global Health Practice and Impact, Georgetown University, Washington, USA; 20 Vanderbilt Institute for Global Health, Vanderbilt University Medical Center, Nashville, TN USA; 21 Elizabeth Glaser Pediatric AIDS Foundation, Washington, USA; 22 University of California San Diego, San Diego, CA USA; 23 United States Agency for International Development, Washington, USA; 24 Fogarty International Center, National Institutes of Health, Bethesda, MD USA; 25 Nigeria Implementation Science Alliance, Abuja, Nigeria

**Keywords:** Mental health, Nigeria, Low and middle-income countries, HIV, Mental health policy, Health services integration

## Abstract

**Background:**

In Nigeria, there is an estimated 1.9 million people living with HIV (PLHIV), 53% of whom utilize HIV care and services. With decreasing HIV-related deaths and increasing new infections, HIV with its associated comorbidities continue to be a key public health challenge in Nigeria. Untreated, comorbid mental disorders are a critical but potentially modifiable determinant of optimal HIV treatment outcomes.

This study aimed to identify the challenges and opportunities related to integrating mental health care into existing HIV programs in Nigeria.

**Method:**

Attendees at the Nigeria Implementation Science Alliance (NISA)‘s 2019 conference participated in nominal group technique (NGT) exercise informed by the “Exploration, Preparation, Implementation, and Sustainment (EPIS)” framework. The NGT process was conducted among the nominal groups in two major sessions of 30-min phases followed by a 30-min plenary session. Data analysis proceeded in four steps: transcription, collation, theming and content analysis.

**Results:**

The two major theoretical themes from the study were – opportunities and challenges of integrating mental health treatment into HIV services. Three sub-themes emerged on opportunities: building on health care facilities for HIV services *(screening, counseling, task-sharing monitoring and evaluation frameworks)*, utilizing existing human resources or workforce in HIV programs *(in-service training and including mental health in education curriculum)* and the role of social and cultural structures *(leveraging existing community, traditional and faith-based infrastructures).* Four sub-themes emerged for challenges: double burden of stigma and the problems of early detection *(HIV and mental health stigma, lack of awareness)*, existing policy gaps and structural challenges *(fragmented health system)*, limited human resources for mental health care in Nigeria *(knowledge gap and burnout)* and dearth of data/evidence for planning and action *(research gaps)*.

**Conclusions:**

Potential for integrating treatments for mental disorders into HIV programs and services exist in Nigeria. These include opportunities for clinicians’ training and capacity building as well as community partnerships. Multiple barriers and challenges such as stigma, policy and research gaps would need to be addressed to leverage these opportunities. Our findings serve as a useful guide for government agencies, policy makers and research organizations to address co-morbid mental disorders among PLHIV in Nigeria.

## Background

Human immunodeficiency virus (HIV) infection has continued to be a global public health problem. Of the 37.9 million people living with HIV (PLHIV) globally, 70% reside in sub-Saharan Africa which has a disproportionate share of new infections (64%) and Acquired Immune Deficiency Syndrome (AIDS)-related deaths (61%) [1]. In Nigeria, there was an estimated 1.9 million PLHIV in 2018, 53% of whom utilize HIV care and services [2, 3]. AIDS-related deaths have decreased in Nigeria by 26% since 2010, and new infections have increased by 5% [3], thereby increasing the population of PLHIV. This increasing population of PLHIV suggests that HIV with its associated comorbidities will continue to be a key public health challenge in Nigeria in future years [4].

The link between HIV infection and mental disorders has been well established. PLHIV are three times more likely than the general population to have mental disorders [5]. The psychosocial impact of HIV and neurologic complications of the infection or of treatment with antiretroviral drugs can increase the risk of mental disorders among PLHIV [6, 7]. On the other hand, individuals with mental disorders are more likely to engage in risky behaviors which can predispose them to HIV infection and increase their chances of transmitting the virus [8]. Depression is the most common mental disorder occurring among PLHIV with prevalence ranging from 9 to 32% in sub-Saharan Africa [5]. In Nigeria, among PLHIV, the prevalence rate is 2.3% for suicide attempt, 2.9% for suicide ideation, 7% for alcohol abuse and 28.2% for major depression [9]. Overall, the prevalence of mental disorders among PLHIV is higher than that of other chronic diseases [10]. Mental disorders affect the HIV care continuum, including delayed initiation of antiretroviral treatment (ART), poor ART adherence [11, 12], poor retention in care [13], accelerated disease progression [14] and low rates of viral suppression. With these effects on the HIV cascade, untreated mental disorders are a critical but potentially modifiable determinant of optimal HIV treatment outcomes [15].

A meta-analysis of 29 studies showed that treatment of depression significantly improved ART adherence [16]. A pilot study of depression treatment for PLHIV in Cameroon showed that all HIV clinical outcomes improved at 4 months after depression treatment [17]. Yet, most people with mental disorders in Nigeria, including PLHIV, have little or no access to mental health treatment [18, 19]. To address this barrier, a key recommendation of the International epidemiology Databases to Evaluate AIDS (IeDEA) consortium is the evaluation of promising models of integrated mental health and HIV care [20]. Several integration models have been developed and tested for both HIV and non-HIV populations. Different integrative, task-sharing approaches involving lay health workers have been used to deliver antidepressants in Cameroon [17], to provide psychosocial interventions to PLHIV in South Africa [14], for problem solving therapy for depression and common mental disorders in Zimbabwe [21], and to improve lay health workers’ knowledge and self-efficacy in managing mental disorders in Malawi [22]. The integrated care approach is a promising, acceptable and feasible strategy for improving both mental health and HIV treatment outcomes in low and middle-income countries (LMIC) [23–25]. However these approaches to integrate HIV and mental health services remain limited by research gaps in cost-effectiveness and long-term impact of different models on patient outcomes and by barriers to their adoption in LMIC [25]. Implementation scientists are well positioned to identify the challenges and opportunities in LMIC for integration of mental health services into existing HIV programs.

The Nigeria Implementation Science Alliance (NISA) was established in 2015 as a robust partnership of 20 organizations that includes academic and non-governmental organizations, clinical service providers, and policy makers involved in HIV care in Nigeria [26]. NISA is aimed at facilitating collaboration among partners, bridging research-to-practice gaps through application of implementation science approaches and implementation research in Nigeria and other countries in Sub-Saharan Africa, and identifying feasible and culturally appropriate strategies for improving public health through research [26, 27]. Yearly, NISA conferences have focused on “Prevention of mother to child HIV transmission (PMTCT)” (2015), “HIV/AIDS and related infections, adolescents health, STI & Hepatitis B” (2016), “Improving Health through Implementation Science & Research” (2017) and “Evidence-based approaches to enhance quality of care” (2018, 26–28). The fifth NISA conference in September 2019 focused on achieving impact through implementation research. It included a nominal group technique (NGT) session with researchers, policy makers, and program implementers focused on the opportunities and challenges for integrating mental health into HIV programs in Nigeria. In this paper, we present the challenges and opportunities identified through the NGT session related to integrating mental health care into existing HIV programs in Nigeria.

## Methods

### Process

We used a modified NGT approach to identify challenges and potential opportunities for integrating evidence-based mental health interventions into HIV services in Nigeria. Participants were drawn from attendees of the NISA 2019 conference and included program staff, researchers, government staff, administrators, donor community, and clinical caregivers. NGT is a structured approach for group “brainstorming”, and it is useful for gathering sets of ideas, information, or recommendations and their relative importance from relevant stakeholders. The NGT process ensures that stakeholders are able to efficiently rank their opinions and perspectives about issues being discussed [28, 29]. In this study, the process of ranking responses helped NISA to identify the priority areas regarding the opportunities and challenges of integrating mental health care into HIV treatment in Nigeria. The duration of the NGT process for this study was 90 min and it was facilitated by five resource persons experienced in NGT [26, 30].

Before commencing the process, the lead facilitator introduced the topic to the participants and highlighted why it was an important public health issue. He stated the purpose of the research and also explained the modified NGT process that was adopted. The NGT process was conducted in two major 30-min sessions, followed by a 30-min plenary session. These three distinct components were designed to engage participants. In the first session, individual group members sequentially identified, discussed and ranked potential challenges and barriers to integrating mental health interventions into existing HIV clinical services and programs in Nigeria. This was followed by group discussion about the identified challenges and barriers. Then, the group voted and ranked these challenges and barriers in order of perceived importance. In the second session, individual group members identified, listed, discussed and ranked the opportunities and strategies for integrating mental health interventions into established HIV clinical services and programs. This was again followed by a group discussion of the lists, another round of voting, and ranking. In both sessions of the NGT process, the group members engaged in collective generation of ideas, discussion, consensus building, and ranking of the challenges of and opportunities for integrating mental health treatment into HIV services in Nigeria. For the plenary discussion, each group selected a lead and rapporteur to moderate and document the group exercise, respectively.

### Analysis

Using a modified NGT analysis approach [31], the data analysis proceeded in four steps [32] Data from each of the nominal groups were individually transcribed and collated per group on Excel™ sheets. Then the multiple-group data was collated to provide the context and background for understanding the ranked-consensus data [33]. The top five-ranked consensus items from each of the groups were then identified and sorted into themes. Within two major categories of (1) challenges with and (2) opportunities for integrating mental health treatment into HIV services, sub-themes were identified through the content analysis process. All data collection documents were de-identified prior to analysis. Ethical approval was obtained for this study from the National Health Research Ethics Committee. Verbal consent (deemed appropriate by the Ethics Committee as this was a focus group study that posed minimal risk to participants) was obtained from the participants and documented in writing.

## Results

Eighty people participated in the NGT process, constituting 11 groups of 6–11 individuals per group. The participants included: 41 program staff, 13 researchers, 10 government staff, 9 administrators and 7 clinical caregivers. The majority of participants had been in their positions for at least 5 years (*n* = 53) **(**Table 1**).**

**Table 1:** Role and experience of the NGT Participants

Job Role	Years of Experience			***n = 80***
Program staff	< 5	5–10	> 10	
	14	18	9	41
Administrator	4	3	2	9
Clinical Care	1	3	3	7
Government Staff	4	3	3	10
Researcher/Academic	4	6	3	13

There were a total of 105 ranked responses for both themes; 53 for opportunities and 52 for challenges. These responses were coalesced (Tables 2 and 3**)** according to ranking by the groups. Three sub-themes emerged from a content analysis of the ranked responses on the opportunities and four sub-themes emerged from a content analysis of the ranked responses for challenges **(**Table 4**)**. These sub-themes are described below.

**Table 2:** Ranking of multi-group outputs of opportunities for integrating mental health services into HIV/AIDS care

Groups	Ranked List of Opportunities
**Group 1**	1. Integrating mental health care into existing HIV screening/testing and counselling 2. Including mental health outcomes into HIV/AIDS progress reports 3. Existing support groups, screening tools and data 4. Availability of National Task-shifting policy 5. Existing counselling session and psychosocial support on STIs, mental health 7. Existing human resource, infrastructure and services
**Group 2**	1. School-based and in-service training of health workers 2. Including mental health integration to HIV/AIDS in funding proposals 3. Revision of existing HIV treatment policy to include mental health 4. Optimizing HIV training opportunities for mental health program 5. Existing policy in support of mental health 6. Existing skilled health workers (Doctors, nurses and counsellors). 7. Clinical evaluation of PLWHA Mental health assessment in HTS services
**Group 3**	1. Leveraging existing traditional and faith-based structures to create awareness on mental health 2. Using existing frameworks for monitoring and evaluation 3. Referrals from ART clinics to Mental Health Department and vice-versa
**Group 4**	1. Integrating ‘mental health basic questions during routine patient care 2. Presence of established referral systems within and between facilities and mental health professionals 3. Engaging faith-based organizations and traditional leaders 4. Prior integration experience; available and provides evidence for integration of mental health intervention 5. Inclusion of mental health in the training curriculum of health workers 6. Existing commitment to HIV elimination 7. Existing support for HIV/Mental health awareness through community partners
**Group 5**	1. Screening for mental health during provision of ANC, PMTCT, Postpartum, Adolescent and Young Adult services 2. Services are available in one place 3. Growing awareness of mental health issues provides an opportunity for integration 4. Attraction for more research and program grants for mental health and HIV/AIDS 5. Support groups implementing mental health education into adolescent friendship centers
**Group 6**	1. Integrating mental health care into patient follow-up/tracking 2. Availability of adolescents and youth clubs in the communities aligned with HIV services

**Table 3:** Ranking of the multi-group outputs of challenges for integrating mental health services into HIV/AIDS care

Groups	Ranked List of Challenges
**Group 1**	1. Poor awareness of mental health by individuals, health care providers, and communities (including detection of early signs) 2. Double stigma’ (HIV and Mental Health) 3. Absence of a National Health Policy on Mental Health in HIV/AIDS 4. Inadequate capacity and/or human resources for mental health care 5. Absence of policy on service integration, integrating mental health and HIV/AIDS
**Group 2**	1. Cultural beliefs about negative spiritual connotations of mental disorders 2. Poor funding for mental health services 3. Absence of national strategic plan on mental health issues, guidelines 4. Inadequate human resources for health care 5. Inadequate number of skilled health workers to offer mental health services 6. Sociocultural barriers like culture and religion 7. Lack of willingness of staff to take on mental health work
**Group 3**	1. Limited capacity and work overload for existing health workers 2. Difficulty in assessing mental health care in the existing system 3. Poor knowledge and skill of health care workers to provide mental health care services 4. Lack of government and donor support for mental health services 5. Poor social care system 6. Poor health-seeking behaviour on mental health issues 10. Knowledge gap from both health care providers and clients 11. Stigmatization of HIV patients
**Group 4**	1. Lack of structures/facilities for mental healthcare management 2. Fragmentation of different units and functions 3. Lack of mental health specific funding 5. Research gap in mental health 6. Affordability of mental health care
**Group 5**	1. Inadequate number of healthcare workers 2. Weak health systems to support mental health care 4. Data paucity for informed decision at the facility level 5. Lack of political will to implement policies 6. Lack/Inadequate policies/guidelines/protocols and guidelines for mental health 7. Getting the buy-in of policy makers 8. Cultural and religious beliefs about mental health
**Group 6**	1. High cost of mental health care

**Table 4:** Themes and sub-themes for integrating mental health care into HIV/AIDS care

Major themes	Sub-themes
Opportunities	1. Building on research and health care facilities for HIV services 2. Utilizing Existing Manpower 3. The role of social and cultural structures
Challenges	1. The Double Burden of Stigma and the Problems of Early Detection 2. Existing Policy Gaps and structural challenges 3. Poor Human Resources for Mental Health Care in Nigeria 4. Dearth of Research for Data and Action

Sub-themes on opportunities for integrating mental health care into HIV/AIDS Services.

### Building on health care facilities for HIV services

There was consensus on the robustness of existing structures around HIV/AIDS services that can be leveraged to integrate mental health care for PLHIV. Specifically, participants identified the HIV pre-test screening/testing counselling process, the task-shifting approach, the existing health screening tools, clinic counselling and mental health assessment during ART clinics as opportunities for leveraging existing HIV services framework for integrating mental health treatment. One participant noted:

*“HIV treatment and care has already been integrated into general out-patient care.” (Clinical care expert/More than 5 years’ experience).*

The existing Monitoring and Evaluation (M&E) frameworks for HIV care in Nigeria were also perceived by participants as potentially useful for assessing effectiveness of any integration of mental health care into HIV services for PLHIV.

### Utilizing existing human resources or workforce in HIV programs

Participants believed that existing human resources for HIV services provide an opportunity for mental health capacity building. This can be achieved through in-service training for HIV health workers, inclusion of mental health care in the training curriculum of health workers and focusing on beneficial impact of mental health treatment on HIV outcomes like improved treatment retention, adherence and viral load suppression among PLHIV. To achieve the need to train existing staff to undertake mental health services, one participant for example suggested that:

*“health workers should be trained rigorously, and strategies put in place with help from patrons.” (Program staff/5–10 years’ experience).*

This is an important approach to leveraging the existing human resources.

### The role of social and cultural structures

Another sub-theme that was identified was the potential for leveraging existing traditional and faith-based infrastructures to create awareness about mental health. The role of the cultural system in ensuring that community members are well aware of and support the mental health of PLHIV is an important component of program integration. Therefore, it is valuable to engage the cultural structures of each community to support integration efforts. A participant noted:

*“use the cultural systems to develop community awareness.” (Program staff/Greater than 10 years’ experience).*

In addition, mental health integration into HIV care can be achieved through community support groups aligned with ffaith-based organizations and traditional leaders. Participants believed that these community support groups can work effectively with HIV service providers and their implementing partners to increase mental health awareness among PLHIV. Support groups can also provide education about mental health in *adolescent friendship centers* that already serve PLHIV. Furthermore, aaccess to mental health care for PLHIV could be improved if social and cultural structures functioned alongside a hypothetically integrated referral system, from ART clinics to existing mmental hhealth services.

Sub-themes on challenges with integrating mental health care into HIV/AIDS Services.

### The double burden of stigma and the problems of early detection

Participants agreed that sstigma thrives on poor awareness and that for PLHIV who experience mental disorders, there is the double burden of stigma arising from HIV and mental illness. Additionally, participants reported that sstigma occurs among PLHIV, health care providers and communities with a culturally driven belief and negative spiritual connotation associated with mental illness. Thus, there is poor mental health awareness among both health care providers and patients, leading to challenges with detecting early signs of mental illness among PLHIV and poor-health seeking behavior. One participant reported that:

*“High level of stigma - mental health and HIV … regular client [HIV patient] contact with health system, provides opportunities for mental health assessment and intervention.” (Program staff/Less than 5 years’ experience).*

### Existing policy gaps and structural challenges

A sub-theme emerged around policy gaps in integrating mental health care into HIV services in Nigeria. Participants identified the absence of service integration plans, national health policy on mental health, national strategic plan on mental health and guideline for monitoring and evaluation tools as major policy gaps posing challenges to integrating mental health into HIV services. A participant noted:

*“lack of policy to drive integration of mental health interventions into HIV programs.” (Program staff/Less than 5 years experience).*

The participants also believed that the existing structures for mental health in Nigeria are insufficient for the established need for mental health care. The operation and organization of mental health care within Nigeria’s current health system are fragmented (operate at only the secondary and tertiary levels of care) and this limits the integration of mental health care into the existing HIV/AIDS care structure (which starts from the primary care level).

### Limited human resources for mental health Care in Nigeria

Participants identified excessive workload for healthcare workers and scarcity of mental health specialists. This occurs because there is limited number of trained mental health workers, coupled with the increasing rate of mental health challenges in the population and among HIV patients. One clinician participant noted:

*“There are few health care workers trained on mental health and there is an increasing incidence of mental health challenges in PLHIV.” (Clinical care/5–10 years’ experience).*

These are potential challenges to integrating mental health care into HIV programs. There was also a perceived knowledge gap among care providers related to mental health that reduces their willingness to take on mental health duties. Furthermore, participants believed that there is burn-out associated with working in mental health care.

### Dearth of data/evidence for planning and action

Finally, a fourth sub-theme emerged about research gaps in mental health in Nigeria. Participants believed that this was a result of poor funding for research in mental health and for infrastructure:

*“specific funding for mental health is lacking.” (Program staff/5–10 years’ experience).*

*“Mental health is not a major focus for HIV programs; thus, donors do not have any mental health indicators to measure.” (Program staff/Less than 5 years’ experience).*

These research gaps lead to limited data that can inform the design and implementation of mental health policies, programs and actions. A participant noted:

*“Few research/work on PLHIV and mental health.” (Clinical care/5–10 years’ experience).*

## Discussion

This NGT exercise with key stakeholders in HIV services in Nigeria and the subsequent analysis identified seven key sub-themes regarding challenges to and opportunities for integrating mental health treatment into existing HIV services for PLHIV in Nigeria. Themes identified can readily be organized in accordance with the Exploration, Preparation, Implementation, and Sustainment (EPIS) framework [34, 35]. There were three themes that emerged for opportunities (*Building on research and health care facilities for HIV services, Utilizing eexisting workforce and leveraging social and cultural structures).* Building on existing service infrastructure and service workforce such as primary health care centers, community youth organizations and faith-based institutions, invokes the EPIS construct of inner organizational context while social and cultural structures are represented in outer context at the country or region level. This finding is important not only for the potential for integrating mental health into HIV programs, but also highlights the feasibility of scale-up and sustainability. In sub-Saharan Africa there are promising examples of specific mental health interventions integrated into HIV/AIDS treatment. In Malawi Udedi et al. integrated depression screening and management into HIV primary care [36]. In Zimbabwe, Duffy et al. described a stepped-care model integrating mental health screening and referral to treatment [37] and in Uganda, Mpungu et al., reported on group psychotherapy for depression integrated into HIV treatment [38]. Some of the structures identified in our study and those utilized in Malawi, Zimbabwe and Uganda are already connected to HIV services infrastructure, and are very widely spread across Nigeria both in rural and urban settings [39, 40].

Four themes were identified relating to challenges and barriers to integration; (*the double burden of HIV and mental health stigma and the problems of early detection, existing health policy gaps and structural challenges, poor human resources for mental health care in Nigeria and dearth of research for data and action).* Issues of stigma and health policy fall within the EPIS outer context domain while early detection and human resources are typically in the inner context domain. These themes reflect cultural and structural barriers that generally affect health care in Nigeria and are consistent with the challenges which confront the process of integrating mental health care into HIV/AIDS treatment in similar settings like Cameroon [41] and Uganda [42]. They also highlight the need to focus on community engagement, public education, and advocacy as part of any intervention geared towards integration of mental health treatments into community HIV programs [43]. These opportunities and challenges may also be better understood in the context of the overall healthcare environment in Nigeria (underfunded, under-resourced and poorly developed), the nature of public-private partnerships that promote health research, sustainability of donor-driven program implementation, and the structure of research frameworks established by existing legislation [44]. Utilizing implementation science frameworks such as EPIS can help organize and consider implementation barriers, facilitators, determinants, mechanisms, and outcomes in outer and inner contexts and the mechanisms that bridge these contexts such as leadership, policies, collaborations, community-academic partnerships and funding [35, 45].

This study involved multi-disciplinary groups of frontline staff, administrators, researchers, and clinicians with at least 5 years’ experience working in HIV programs and services in Nigeria. Thus, their perceptions and experiences are relevant to understanding these potential opportunities and barriers to integrating mental health treatments into HIV programs. Further, the varied perspectives and NGT process allowed for representation of EPIS implementation factors including outer context, inner context, bridging factors, interorganizational relationships, and innovation characteristics. Based on self-reported roles, most participants had direct knowledge and experience about the context of HIV care, services and program implementation in Nigeria. They also understood the role of government and funding agencies, the policy framework supporting the programs and the funding streams available in Nigeria for healthcare research. In addition, utilizing the multi-disciplinary group of professionals facilitated meaningful brainstorming from multiple perspectives and the generation of practical ideas.

Integrating mental health into primary health care and other non-specialty health settings is the bedrock of Nigeria’s policy on mental health access [46]. Unfortunately, due to inadequate funding, limited mental health specialists and lack of legislative framework, this stated policy has not translated into state or federal laws or clinical practice [47]. This may explain the perception by participants of lack of policy frameworks for integrating interventions for mental disorders into HIV services. However, there are existing, established tools and frameworks for integrating mental health into non-psychiatric settings using evidence-based task-sharing approaches such as the World Health Organization (WHO)‘s mental health gap action plan (mhGAP) [48]. The mhGAP has already been contextualized and tested in Nigeria [49, 50]. Given the potential for integration of mental health treatments within HIV programs identified in this study and the availability of standardized, validated tools for such integration, efforts should be geared towards pilot implementation studies to explore the feasibility and effectiveness of mental health treatments integrated into HIV programs and services. NISA’s collaborating organizations and partner government agencies (the Federal Ministry of Health, the National Primary Healthcare Development Agency, and the National Agency for the Control of AIDS) can play a pioneering role in leveraging their relationships, academic partnerships, and funding agencies towards this goal [26, 30].

As identified by participants in this study, stigmatizing beliefs and negative attitudes towards HIV and mental disorders are a major barrier to accessing both mental health and HIV treatments [51, 52]. There is evidence that collaboration between relevant government agencies in Nigeria and local and international non-governmental organizations to develop and implement strategic communication programs helped reduce HIV-related stigma. Exposure to HIV-related communication in the media increased knowledge about HIV and reduced negative attitudes towards people living with HIV [53]. Lessons from such strategy around HIV and HIV-related services can be adapted and included in the design of interventions to integrate mental health treatments into HIV services.

There are limitations of this study that are worth noting. Firstly, the conference was open to staff from all HIV programs in Nigeria but participation was limited to those who were able to pay the required registration fees and available to attend at the conference dates and times. Secondly, the design and format of the NGT exercise, unlike a free-flowing focus group, is rigid and time-limited. To address this limitation, participants were grouped to include a mix of experiences, roles, and educational levels thereby ensuring that the perspectives, ideas and emergent consensus were as representative as possible. Thirdly, as in other group participation-based research, participants’ opinions and ideas may have been influenced by others in the group. To reduce the impact of this group factor, we utilized many small groups instead of few larger groups and identified moderators for each group who ensured that each individual member contributed adequately to the group discussions [54]. Finally, data collection, collation, and analyses were conducted by the authors. This may inadvertently lead to the infusion of the authors’ perspectives in interpreting the data. To address this, we have included authors with varying backgrounds and experiences to ensure balanced perspectives in the analysis and interpretation of the results [55].

## Conclusion

Potential for integrating treatments for mental disorders into HIV programs and services exist in Nigeria. These include opportunities for clinicians’ training and capacity building as well as community partnerships. Multiple barriers and challenges such as funding, stigma and policy gaps would need to be addressed to leverage these opportunities. The subthemes that emerged from this study reflect the on-the-ground experiences, practical realities and collective wisdom of professionals involved in HIV programs and services in Nigeria. Our findings serve as a useful guide for government agencies, policy makers, research organizations and local foundations in Nigeria to address co-morbid mental disorders among PLHIV in Nigeria.

## Data Availability

The datasets used and/or analysed during the current study available from the corresponding author on reasonable request.

## Abbreviations

HIV: Human Immunodeficiency Virus
PLHIV: People Living with HIV
AIDS: Acquired Immune Deficiency Syndrome
ART: Antiretroviral treatment
LMIC: Low and middle-income countries
NISA: Nigeria Implementation Science Alliance
NGT: Nominal Group Technique
EPIS: Exploration, Preparation, Implementation, and Sustainment
WHO: World Health Organization
mnGAP: Mental health Gap Action Plan

## References

[1] Global HIV and AIDS Statistics-2019 fact sheet. 2019 2019.

[2] UNAIDS (2018). Country report-Nigeria.

[3] Nigeria HIV /AIDS Indicator and Impact Survey. Federal Ministry of Health N; 2019 2019.

[4] Frank TD, Carter A, Jahagirdar D, Biehl MH, Douwes-Schultz D, Larson SL (2019). Global, regional, and national incidence, prevalence, and mortality of HIV, 1980–2017, and forecasts to 2030, for 195 countries and territories: a systematic analysis for the global burden of diseases, injuries, and risk factors study 2017. The Lancet HIV.

[5] Bernard C, Dabis F, de Rekeneire N (2017). Prevalence and factors associated with depression in people living with HIV in sub-Saharan Africa: A systematic review and meta-analysis. PLoS One.

[6] Abas M, Ali GC, Nakimuli-Mpungu E, Chibanda D (2014). Depression in people living with HIV in sub-Saharan Africa: time to act. Tropical Med Int Health.

[7] Kranick SM, Nath A (2012). Neurologic complications of HIV-1 infection and its treatment in the era of antiretroviral therapy. CONTINUUM: Lifelong Learning in Neurology.

[8] Yun LW, Maravi M, Kobayashi JS, Barton PL, Davidson AJ (2005). Antidepressant treatment improves adherence to antiretroviral therapy among depressed HIV-infected patients. JAIDS.

[9] Egbe CO, Dakum PS, Ekong E, Kohrt BA, Minto JG, Ticao CJ (2017). Depression, suicidality, and alcohol use disorder among people living with HIV/AIDS in Nigeria. BMC Public Health.

[10] Abiodun O, Lawal I, Omokanye C (2018). PLHIV are more likely to have mental distress: evidence from a comparison of a cross-section of HIV and diabetes patients at tertiary hospitals in Nigeria. AIDS Care.

[11] Gonzalez JS, Batchelder AW, Psaros C, Safren SA (2011). Depression and HIV/AIDS treatment nonadherence: a review and meta-analysis. J Acquir Immune Defic Syndr (1999).

[12] Adeoti AO, Dada M, Elebiyo T, Fadare J, Ojo O. Survey of antiretroviral therapy adherence and predictors of poor adherence among HIV patients in a tertiary institution in Nigeria. Pan Afr Med J. 2019;33. 10.11604/pamj.2019.33.277.18711 PMC6815489 31692880

[13] Smillie K, Borek NV (2014). Kop MLvd, Lukhwaro a, li N, Karanja S, et al. Mobile health for early retention in HIV care: a qualitative study in Kenya (WelTel retain). Afr J AIDS Res.

[14] Petersen I, Hancock JH, Bhana A, Govender K (2014). A group-based counselling intervention for depression comorbid with HIV/AIDS using a task shifting approach in South Africa: a randomized controlled pilot study. J Affect Disord.

[15] Parcesepe AM, Mugglin C, Nalugoda F, Bernard C, Yunihastuti E, Althoff K (2018). Screening and management of mental health and substance use disorders in HIV treatment settings in low-and middle-income countries within the global Ie DEA consortium. J Int AIDS Soc.

[16] Sin NL, DiMatteo MR (2014). Depression treatment enhances adherence to antiretroviral therapy: a meta-analysis. Ann Behav Med.

[17] Gaynes BN, Pence BW, Atashili J, O’Donnell JK, Njamnshi AK, Tabenyang ME (2015). Changes in HIV outcomes following depression care in a resource-limited setting: results from a pilot study in Bamenda, Cameroon. PLoS One.

[18] Demyttenaere K, Bruffaerts R, Posada-Villa J, Gasquet I, Kovess V, Lepine J (2004). Prevalence, severity, and unmet need for treatment of mental disorders in the World Health Organization world mental health surveys. Jama..

[19] Wang PS, Aguilar-Gaxiola S, Alonso J, Angermeyer MC, Borges G, Bromet EJ (2007). Use of mental health services for anxiety, mood, and substance disorders in 17 countries in the WHO world mental health surveys. Lancet.

[20] Parcesepe AM, Bernard C, Agler R, Ross J, Yotebieng M, Bass J (2018). Mental health and HIV: research priorities related to the implementation and scale up of ‘treat all’in sub-Saharan Africa. J Virus Erad.

[21] Chibanda D, Mesu P, Kajawu L, Cowan F, Araya R, Abas MA (2011). Problem-solving therapy for depression and common mental disorders in Zimbabwe: piloting a task-shifting primary mental health care intervention in a population with a high prevalence of people living with HIV. BMC Public Health.

[22] Wright J, Common S, Kauye F, Chiwandira C (2014). Integrating community mental health within primary care in southern Malawi: a pilot educational intervention to enhance the role of health surveillance assistants. Int J Soc Psychiatry.

[23] Sikkema KJ, Dennis AC, Watt MH, Choi KW, Yemeke TT, Joska JA. Improving mental health among people living with HIV: a review of intervention trials in low-and middle-income countries. Global Mental Health. 2015;2. 10.1017/gmh.2015.17 PMC4589870 26435843

[24] Kulisewa K, Stockton MA, Hosseinipour MC, Gaynes BN, Mphonda S, Udedi MM (2019). The role of depression screening and treatment in achieving the UNAIDS 90–90–90 goals in sub-Saharan Africa. AIDS Behav.

[25] FLH C, Haldane VE, Cervero-Liceras F, Ong SE, Sigfrid LA, Murphy G (2017). Interventions and approaches to integrating HIV and mental health services: a systematic review. Health policy and planning.

[26] Ezeanolue EE, Powell BJ, Patel D, Olutola A, Obiefune M, Dakum P (2016). Identifying and prioritizing implementation barriers, gaps, and strategies through the Nigeria implementation science alliance: Getting to zero in the prevention of mother-to-child transmission of HIV. J Acquir Immune Deficiency Syndr (1999).

[27] Ezeanolue EE, Menson WNA, Patel D, Aarons G, Olutola A, Obiefune M (2018). Gaps and strategies in developing health research capacity: experience from the Nigeria implementation science Alliance. Health research policy and systems.

[28] Harvey N, Holmes CA (2012). Nominal group technique: an effective method for obtaining group consensus. Int J Nurs Pract.

[29] McMillan SS, King M, Tully MP (2016). How to use the nominal group and Delphi techniques. Int J Clin Pharm.

[30] Ezeanolue EE, Iheanacho T, Patel DV, Patel S, Sam-Agudu N, Obiefune M (2019). Challenges and Strategies for Improving Training of Mid-Level Research Personnel in Nigeria. Ann Global Health.

[31] Søndergaard E, Ertmann RK, Reventlow S, Lykke K (2018). Using a modified nominal group technique to develop general practice. BMC Fam Pract.

[32] Van Breda A (2005). Steps to analysing multiple-group NGT data. Soc Work Practitioner-Researcher.

[33] Manera K, Hanson C, Gutman T, Tong A (2018). Consensus methods: nominal group technique. Handbook of Research Methods in Health Social Sciences.

[34] Aarons GA, Hurlburt M, Horwitz SM (2011). Advancing a conceptual model of evidence-based practice implementation in public service sectors. Adm Policy Ment Health Ment Health Serv Res.

[35] Moullin JC, Dickson KS, Stadnick NA, Rabin B, Aarons GA (2019). Systematic review of the exploration, preparation, implementation, sustainment (EPIS) framework. Implement Sci.

[36] Udedi M, Stockton MA, Kulisewa K, Hosseinipour MC, Gaynes BN, Mphonda SM (2018). Integrating depression management into HIV primary care in Central Malawi: the implementation of a pilot capacity building program. BMC Health Serv Res.

[37] Duffy M, Sharer M, Cornman H, Pearson J, Pitorak H, Fullem A (2017). Integrating mental health and HIV services in zimbabwean communities: a nurse and community-led approach to reach the most vulnerable. J Assoc Nurses AIDS Care.

[38] Nakimuli-Mpungu E, Wamala K, Okello J, Alderman S, Odokonyero R, Mojtabai R (2015). Group support psychotherapy for depression treatment in people with HIV/AIDS in northern Uganda: a single-Centre randomised controlled trial. Lancet HIV..

[39] Ezegbe C, Stephenson N (2012). The reach and limits of the US president’s emergency plan for aids relief (PEPFAR) funding of prevention of mother-to-child transmission (PMTCT) of HIV in Nigeria. Afr J Reprod Health.

[40] Banigbe B, Audet CM, Okonkwo P, Arije OO, Bassi E, Clouse K (2019). Effect of PEPFAR funding policy change on HIV service delivery in a large HIV care and treatment network in Nigeria. PLoS One.

[41] Pence BW, Gaynes BN, Atashili J, O’Donnell JK, Kats D, Whetten K (2014). Feasibility, safety, acceptability, and preliminary efficacy of measurement-based care depression treatment for HIV patients in Bamenda. Cameroon AIDS and Behavior.

[42] Zhong H, Arjmand IK, Brandeau ML, Bendavid E. Health outcomes and cost-effectiveness of treating depression in people with HIV in Sub-Saharan Africa: a model-based analysis. [published online ahead of print, 2020 Jan 27]. AIDS Care. 2020:1–7. 10.1080/09540121.2020.1719966. 10.1080/09540121.2020.1719966 PMC7382984 31986900

[43] Eaton J, Gureje O, De Silva M, Sheikh TL, Ekpe EE, Abdulaziz M (2018). A structured approach to integrating mental health services into primary care: development of the mental health scale up Nigeria intervention (mhSUN). Int J Ment Heal Syst.

[44] Aliyu M, Varkey P, Salihu H, Iliyasu Z, Abubakar I (2010). The HIV/AIDS epidemic in Nigeria: progress, problems and prospects. Afr J Med Med Sci.

[45] Proctor E, Silmere H, Raghavan R, Hovmand P, Aarons G, Bunger A (2011). Outcomes for implementation research: conceptual distinctions, measurement challenges, and research agenda. Adm Policy Ment Health Ment Health Serv Res.

[46] Health FMo. The National Mental Health Policy for Nigeria. In: Health Mo, editor. Lagos1991.

[47] Mugisha J, Abdulmalik J, Hanlon C, Petersen I, Lund C, Upadhaya N (2017). Health systems context (s) for integrating mental health into primary health care in six emerald countries: a situation analysis. Int J Ment Heal Syst.

[48] Organization WH. mhGAP intervention guide for mental, neurological and substance use disorders in non-specialized health settings: mental health Gap Action Programme (mhGAP). 2010. 23741783

[49] Gureje O, Abdulmalik J, Kola L, Musa E, Yasamy MT, Adebayo K (2015). Integrating mental health into primary care in Nigeria: report of a demonstration project using the mental health gap action programme intervention guide. BMC Health Serv Res.

[50] Abdulmalik J, Kola L, Fadahunsi W, Adebayo K, Yasamy MT, Musa E (2013). Country contextualization of the mental health gap action programme intervention guide: a case study from Nigeria. PLoS Med.

[51] Iheanacho T, Marienfeld C, Stefanovics E, Rosenheck RA (2014). Attitudes toward mental illness and changes associated with a brief educational intervention for medical and nursing students in Nigeria. Academic Psychiatr.

[52] Onyebuchi-Iwudibia O, Brown A (2014). HIV and depression in eastern Nigeria: the role of HIV-related stigma. AIDS Care.

[53] Babalola S, Fatusi A, Anyanti J (2009). Media saturation, communication exposure and HIV stigma in Nigeria. Soc Sci Med.

[54] Cyr J (2019). Focus groups for the social science researcher: Cambridge University press.

[55] McMillan SS, Kelly F, Sav A, Kendall E, King MA, Whitty JA (2014). Using the nominal group technique: how to analyse across multiple groups. Health Serv Outcomes Res Methodology.

